# Supplementary material for: Transient neuronal suppression for exploitation of new sensory evidence
Source: Nat Commun. 2022 Jan 10;13:23. doi: 10.1038/s41467-021-27697-4 (PMC8748884; doi:10.1038/s41467-021-27697-4)
Supplement: Supplementary file 1 — Supplementary Information [file 41467_2021_27697_MOESM1_ESM.pdf]

Transient neuronal suppression for exploitation of new sensory evidence

Maxwell Shinn, Daeyeol Lee, John D. Murray, Hyojung Seo

Supplementary information

Supplementary Figure 1

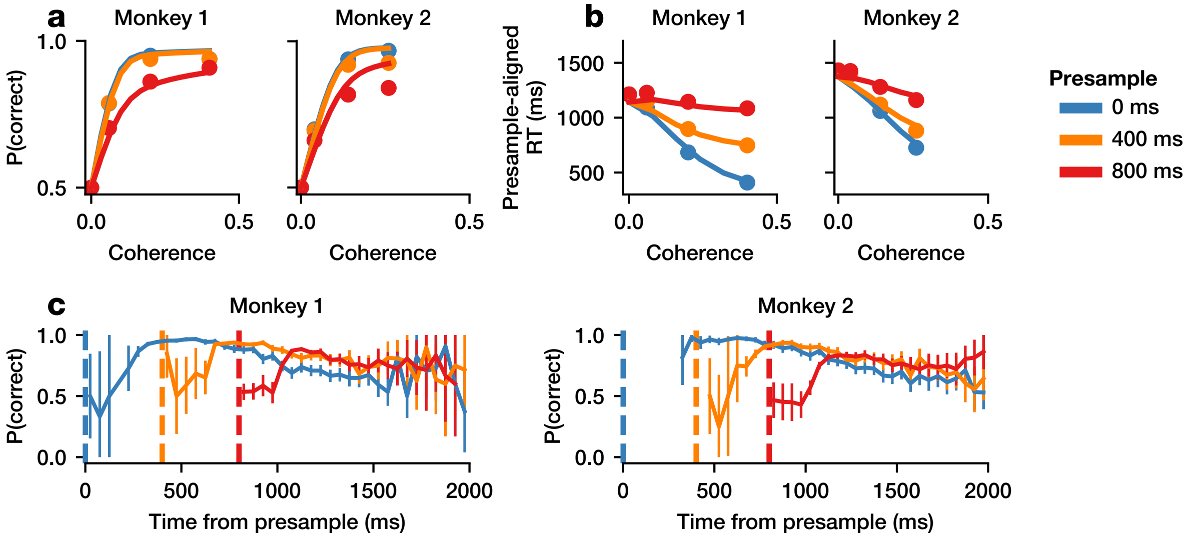

Supplementary Figure 1: Behavioral data and model prediction. (a-b) Psychometric (a) and chronometric (b) functions. Markers indicate data, and lines indicate the predictions of the motor suppression GDDM. Error bars representing 95% confidence interval of the mean are hidden beneath the markers. (c) Psychometric function extended over time in 50 ms bins. Time points are hidden for bins without at least one correct and one incorrect response. Error bars represent 95% confidence interval of the ratio computed with a normal approximation. Analyses are based on 28,378 trials for Monkey 1 and 19,514 trials for Monkey 2.

Supplementary Figure 2

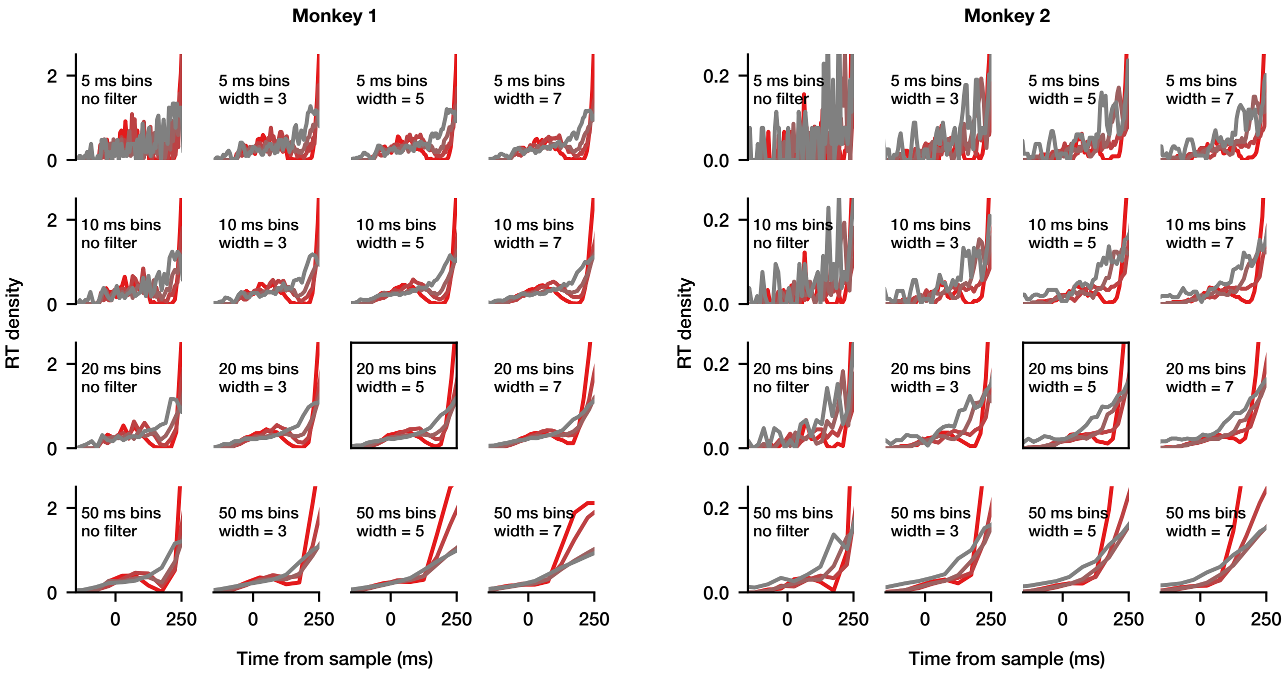

Supplementary Figure 2: RT dip is robust across bin sizes and smoothing widths. For each monkey, we constructed the RT histogram immediately after sample onset for 800 ms presample trials, analogous to Figure 2b,d, with varying bin sizes and smoothing widths, inset in each figure. Smoothing width was defined as the filter width of the order 1 Savitzky-Golay filter. The label "no filter" indicates the absence of smoothing. The box indicates the bin size and filter width chosen for the present study.

Supplementary Figure 3

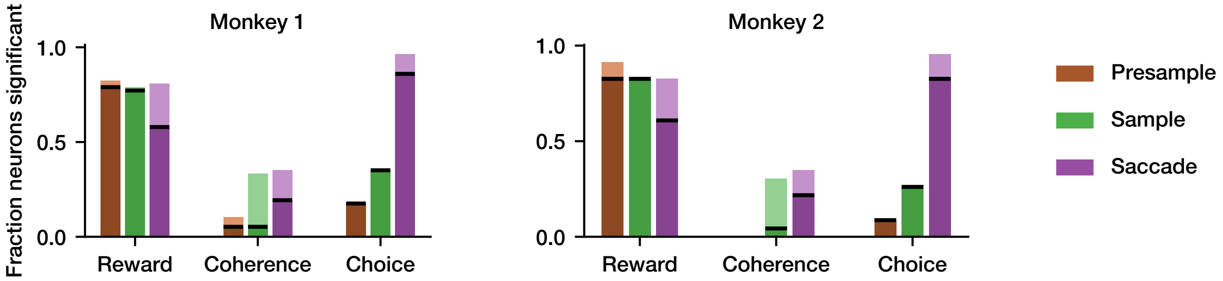

Supplementary Figure 3: Influence of task conditions on firing rate. For each neuron, a regression model was fit to a 100-ms interval at the onset of the presample, sample, and saccade. Each model included four terms—coherence, the large reward target in the response field, the choice into the response field, and an intercept. The number of neurons for which each coefficient showed a significantly positive ($p < 0.05$, two-tailed t-test) value is shown for each monkey. Dark colors indicate a significantly positive value, and light colors indicate a significantly negative value.

Supplementary Figure 4

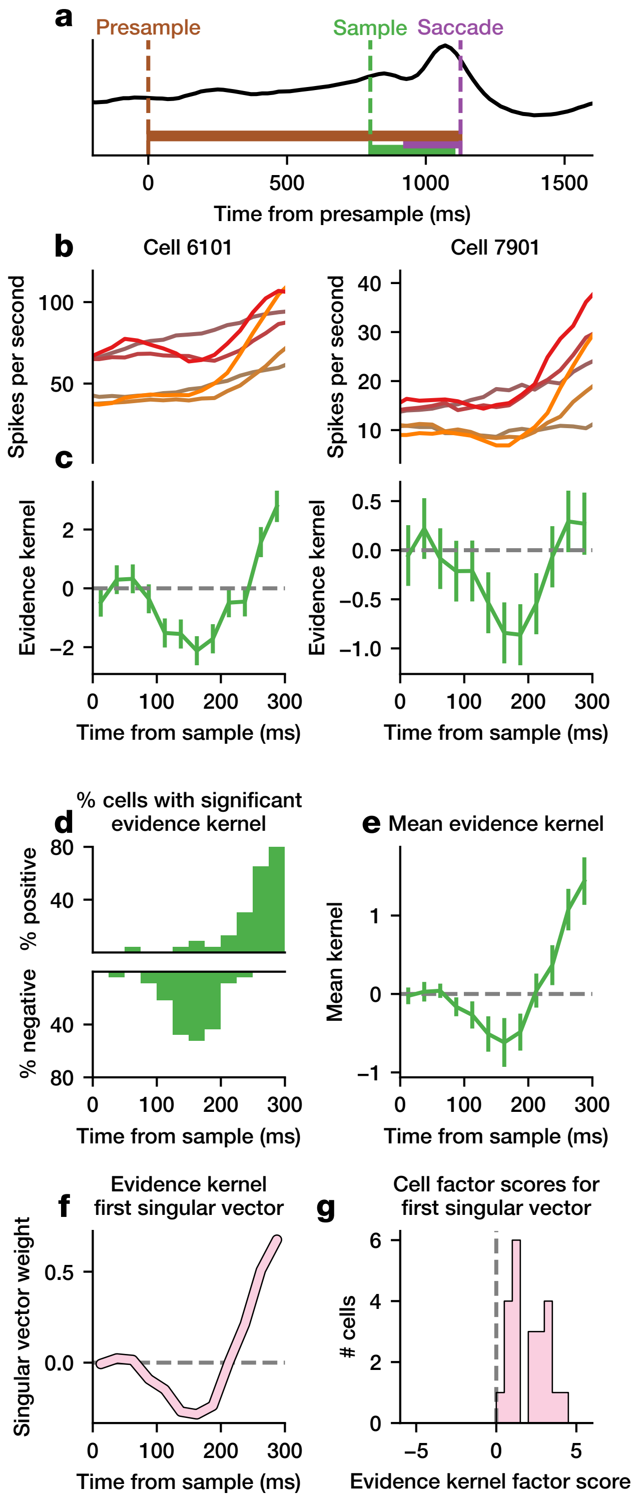

Supplementary Figure 4: Evidence dip in individual FEF neurons for Monkey 2. (a) A schematic of the regression model showing alignment of the kernels to the presample, sample, and saccade. (b) Smoothed firing rate for two example neurons across conditions. Colors are the same as in Figures 2-3. (c) Evidence kernel for the example neurons in (b). Error bars indicate 95% confidence interval of the regression coefficient. (d) For each point in time, the number of neurons in the population with significantly positive (top) or negative (bottom) evidence kernel is shown ($p < 0.05$, one-tailed t-test). (e) Mean evidence kernel across neurons. Error bars indicate 95% confidence interval of the mean. (f) The first singular vector of the evidence kernels is shown, along with (g) the corresponding factor scores of each neuron. Analyses are based on 23 neurons.

Supplementary Figure 5

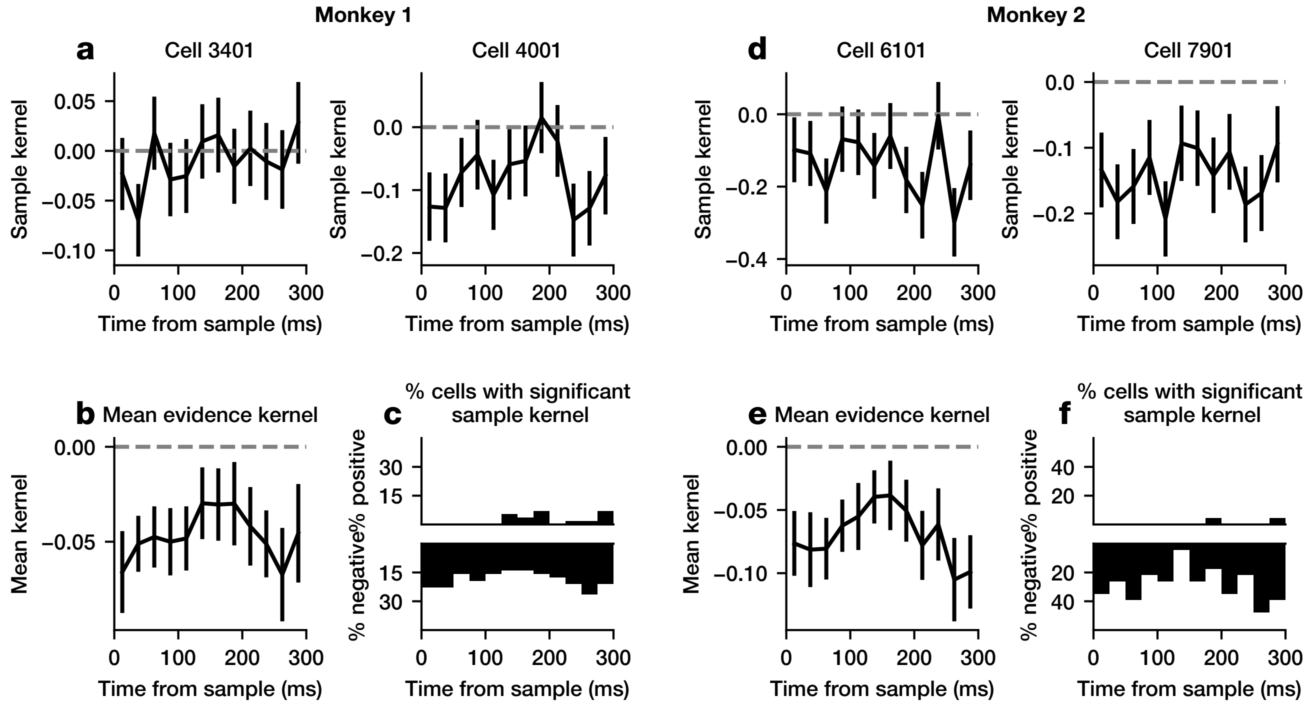

Supplementary Figure 5: The sample kernel (referred to as E kernel in the Methods) does not show a dip. Plots shown for Monkey 1 (a-c) and Monkey 2 (d-f). (a,d) Sample kernel for the example neurons in Figure 4b (Monkey 1) and Supplementary Figure 4b (Monkey 2). Error bars indicate 95% confidence interval of the regression coefficient. (b,e) Mean evidence kernel across neurons. Error bars indicate 95% confidence interval of the mean. (b,e) For each point in time, the number of neurons in the population with significantly positive (top) or negative (bottom) evidence kernel is shown ($p < 0.05$, one-tailed t-test). Analyses are based on 57 neurons for Monkey 1 and 23 neurons for Monkey 2.

Supplementary Figure 6

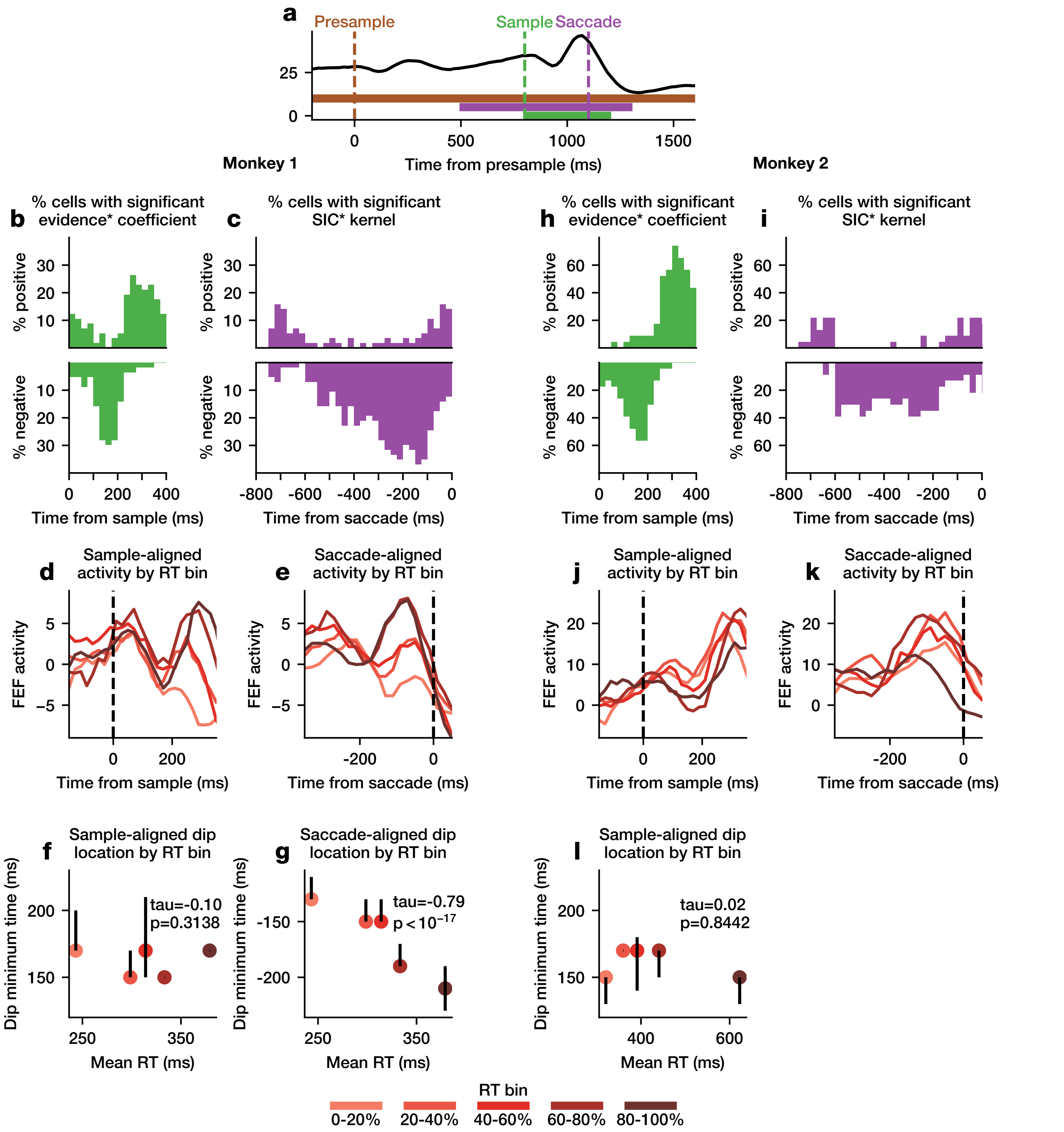

Supplementary Figure 6: The evidence dip is not saccade-related. (a) A schematic of the alternative regression model, with kernels designated by a * suffix. (b,c,h,i) Format similar to Figure 4d, except using the evidence* kernel (b,h) or SIC* kernel (c,i) instead of the evidence kernel, for Monkey 1 (b,c) and 2 (h,i). (d,j) Normalized FEF activity from high-coherence 800 ms presample trials is aligned to the sample onset and plotted separately for each of 5 RT bins for Monkey 1 (d) and 2 (j). (e,k) Normalized FEF activity from these trials is aligned to the saccade onset and plotted separately for each of 5 RT bins for Monkey 1 (e) and 2 (k). (f,l) Time of the first local minimum after the sample onset for the curves in (e) and (k) are plotted for Monkey 1 (f) and 2 (l) against the mean RT of the trials in the RT bin. Points indicate the median of 15 resamplings of the FEF activity, and error bars indicate the interquartile range. Inset is the Kendalls tau correlation r and the corresponding two-tailed p-value across all resamplings. (g) Time of the first local minimum before the saccade onset for the curves in (f) are plotted for Monkey 1 against the mean RT of the trials in the RT bin. Inset is the Kendalls tau correlation r and the corresponding two-tailed p-value across all resamplings. Minima could not be reliably detected in for saccade-aligned activity in Monkey 2.

Supplementary Figure 7

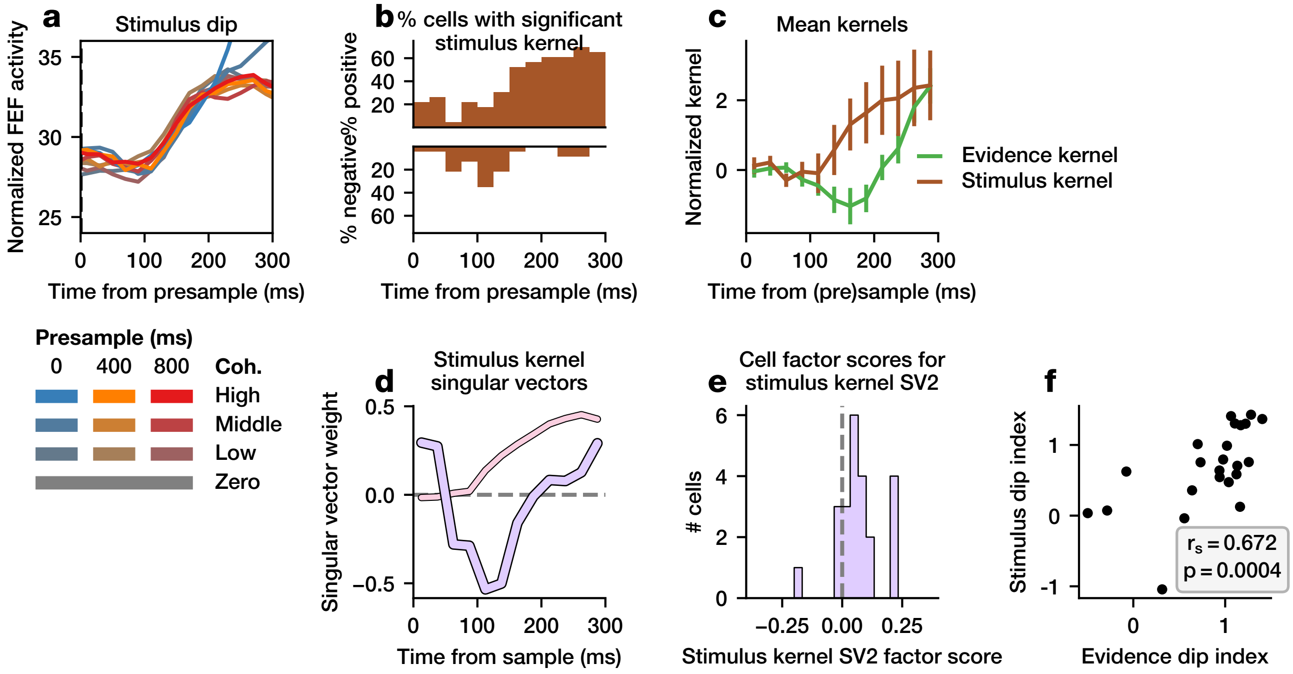

Supplementary Figure 7: Comparison of evidence and stimulus dips for Monkey 2. (a) Mean FEF activity is plotted for each presample and coherence condition at presample (400 or 800 ms presample) or sample (0 ms presample) onset. (b) The fraction of neurons at each point in time with significantly positive (top) or negative (bottom) stimulus kernel ($p < 0.05$, one-tailed t-test). (c) Mean stimulus kernel is shown with overlaid evidence kernel. Time is given as time since the sample (evidence kernel) or presample (stimulus kernel). Error bars indicate 95% confidence interval of the mean. (d) The first (SV1) and second (SV2) singular vector of the stimulus kernels are shown, along with (e) the corresponding factor scores for each neuron on SV2. (f) Evidence dip index is plotted against stimulus dip index. Spearman correlation and two-tailed p-value is inset. Analyses are based on 23 neurons.

Supplementary Figure 8

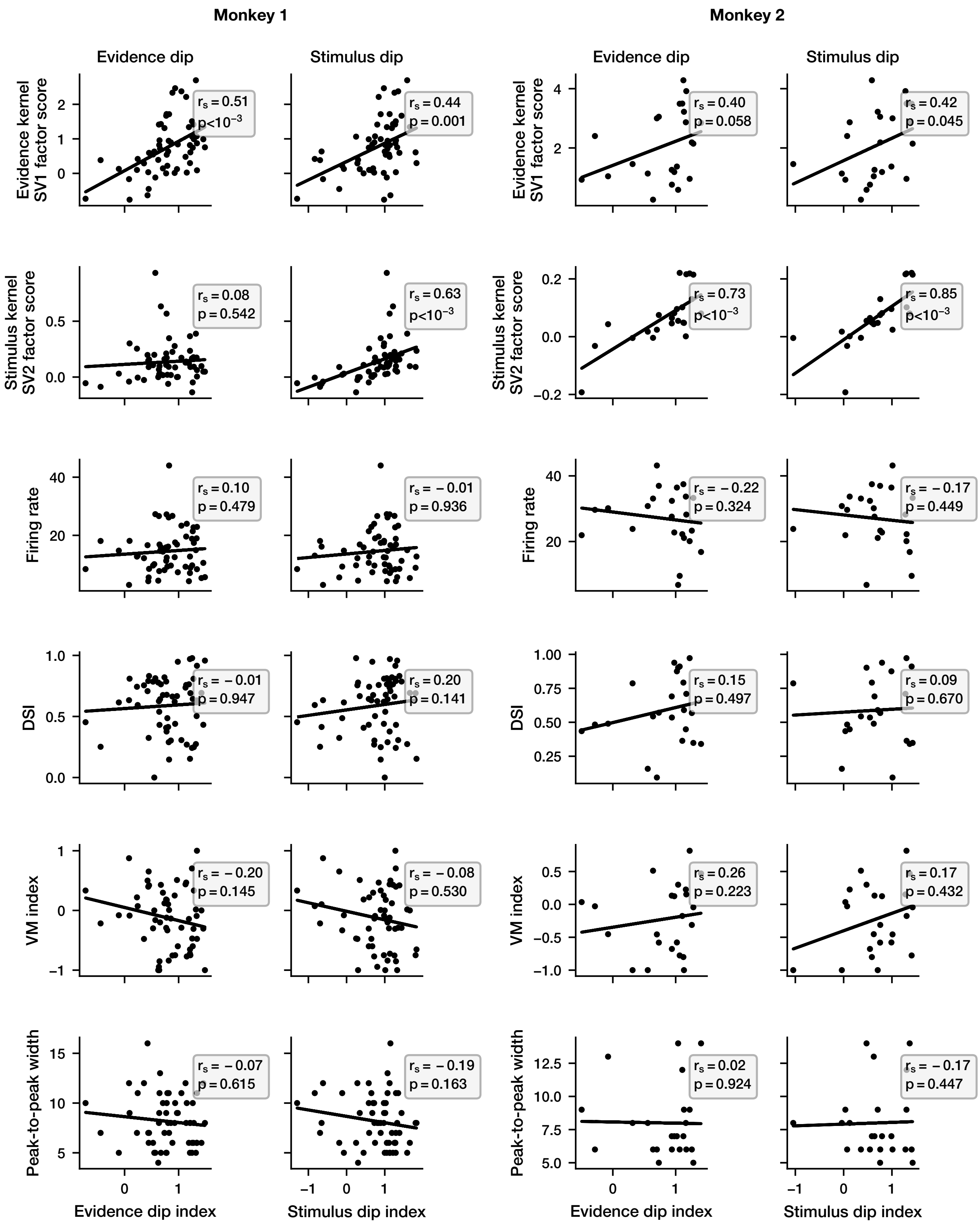

Supplementary Figure 8: Evidence and stimulus dip indices are not correlated with neuron properties. The evidence and stimulus dip indices for each neuron are plotted against the weight on SV2 of the evidence kernel, coefficient of partial determination (CPD) for the evidence kernel, firing rate, directional selectivity index (DSI), visuomovement (VM) index, and the neuron's mean spike peak-to-peak width. Spearman correlation and two-tailed p-value is inset.

Supplementary Figure 9

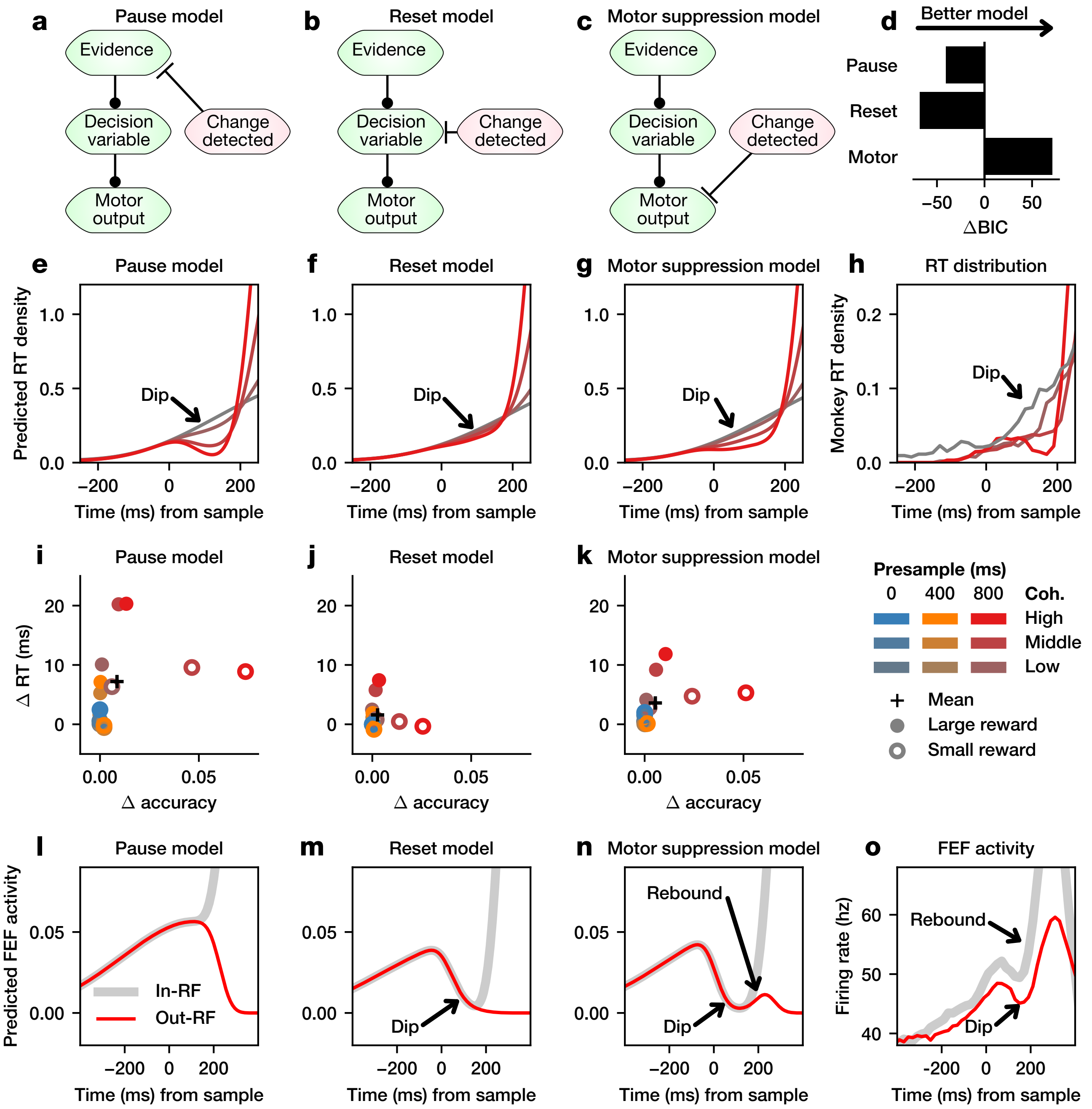

Supplementary Figure 9: Comparison of computational models of the dip for Monkey 2. (a-c) Schematic of the (a) pause model, (b) reset model, and (c) motor suppression model across stages of the decision-making process. (d) The fit of each GDDM to the RT distribution, as quantified by BIC, is shown for each of the three models. (e-g) The simulated RT distribution for 800 ms presample at the time of evidence onset is shown for the (e) pause, (f) reset, and (g) motor suppression models. (h) The RT distribution for Monkey 2 in 800 ms presample trials at the time of evidence onset. (i-k) For each coherence, presample, and reward condition, the difference in mean RT and accuracy, with and without the dip mechanism, are plotted for the (i) pause, (j) reset, and (k) motor suppression models. (l-n) Neural predictions of the (l) pause model, (m) reset model, and (n) motor suppression model, based on the mean decision variable or motor-decision variable (see Methods) for inside (in-RF, black) and outside (out-RF, red) the response field on correct high-coherence trials for models fit to the RT distribution of Monkey 2. (o) Population activity from FEF neurons in Monkey 2 for correct responses inside (gray) and outside (red) the response field for high-coherence, 800-ms presample trials.

Supplementary Figure 10

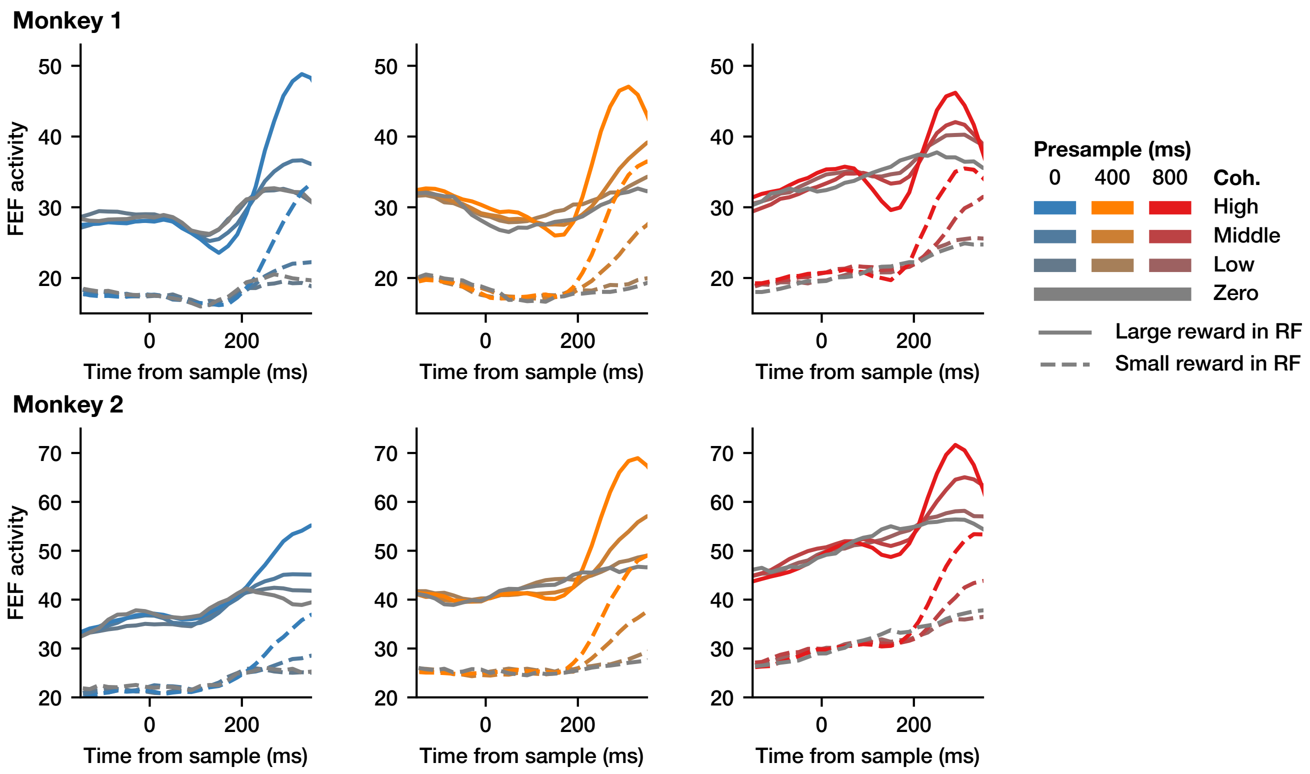

Supplementary Figure 10: Dip magnitude is largest at highest activity. The dip is shown for all presample and coherence conditions for trials where the large or small reward target is inside and outside the response field (RF).
