# Supplementary material for: Transient neuronal suppression for exploitation of new sensory evidence
Source: Nat Commun. 2022 Jan 10;13:23. doi: 10.1038/s41467-021-27697-4 (PMC8748884; doi:10.1038/s41467-021-27697-4)
Supplement: Supplementary file 3 — Reporting Summary [file 41467_2021_27697_MOESM3_ESM.pdf]

nature research

Corresponding author(s): John Murray, Hyojung Seo

Last updated by author(s): 07-26-2021

Reporting Summary

Nature Research wishes to improve the reproducibility of the work that we publish. This form provides structure for consistency and transparency in reporting. For further information on Nature Research policies, see our Editorial Policies and the Editorial Policy Checklist.

Statistics

For all statistical analyses, confirm that the following items are present in the figure legend, table legend, main text, or Methods section.

n/a	Confirmed	
☐	☒	The exact sample size (*n*) for each experimental group/condition, given as a discrete number and unit of measurement
☐	☒	A statement on whether measurements were taken from distinct samples or whether the same sample was measured repeatedly
☐	☒	The statistical test(s) used AND whether they are one- or two-sided<br>*Only common tests should be described solely by name; describe more complex techniques in the Methods section.*
☐	☒	A description of all covariates tested
☒	☐	A description of any assumptions or corrections, such as tests of normality and adjustment for multiple comparisons
☐	☒	A full description of the statistical parameters including central tendency (e.g. means) or other basic estimates (e.g. regression coefficient) AND variation (e.g. standard deviation) or associated estimates of uncertainty (e.g. confidence intervals)
☐	☒	For null hypothesis testing, the test statistic (e.g. *F*, *t*, *r*) with confidence intervals, effect sizes, degrees of freedom and *P* value noted<br>*Give P values as exact values whenever suitable.*
☒	☐	For Bayesian analysis, information on the choice of priors and Markov chain Monte Carlo settings
☒	☐	For hierarchical and complex designs, identification of the appropriate level for tests and full reporting of outcomes
☐	☒	Estimates of effect sizes (e.g. Cohen's *d*, Pearson's *r*), indicating how they were calculated

Our web collection on statistics for biologists contains articles on many of the points above.

Software and code

Policy information about availability of computer code

Data collection	Stimulus presentation was controlled by custom software designed for our experimental setup. Custom software was used for neural recordings and for aligning spikes with stimulus and behavioral data.
Data analysis	Data were analyzed using the standard scientific stack for Python 3. GDDMs were simulated using PyDDM 0.5.0.

For manuscripts utilizing custom algorithms or software that are central to the research but not yet described in published literature, software must be made available to editors and reviewers. We strongly encourage code deposition in a community repository (e.g. GitHub). See the Nature Research guidelines for submitting code & software for further information.

Data

Policy information about availability of data

All manuscripts must include a data availability statement. This statement should provide the following information, where applicable:

- Accession codes, unique identifiers, or web links for publicly available datasets
- A list of figures that have associated raw data
- A description of any restrictions on data availability

GDDMs used in the study are available at: https://pyddm.readthedocs.io/en/latest/cookbook/papers/dip.html. Neurophysiological data is hosted in a local SQL database, and are available from corresponding author by request.

Field-specific reporting

Please select the one below that is the best fit for your research. If you are not sure, read the appropriate sections before making your selection.

- [x] Life sciences
- [ ] Behavioural & social sciences
- [ ] Ecological, evolutionary & environmental sciences

For a reference copy of the document with all sections, see nature.com/documents/nr-reporting-summary-flat.pdf

Life sciences study design

All studies must disclose on these points even when the disclosure is negative.

Sample size	80 neurons (N=57 and 23 from monkeys 1 and 2, respectively) recorded from primate FEF were analyzed. Sample size was determined to include all the known classes of FEF neurons in the dataset, that exhibit distinct/stereotypical response properties to visual stimulus and saccadic eye movement.
Data exclusions	We excluded 44 neurons from Monkey 1 and 15 neurons from Monkey 2, either because a target was not appropriately located in their response field or they were not recorded with a sufficient number of trials (N<50 trials). The criteria were pre-established before any analyses were performed.
Replication	We replicated the analysis performed on Monkey 1 in Monkey 2. As described in the manuscript, all main results were replicated in both monkeys.
Randomization	The study used within-subject design, and experimental conditions (e.g. the location of the high reward and correct target, color coherence, and presample duration) were randomized on a trial-by-trial basis within each animal.
Blinding	Blinding is not relevant to the present study, because there was no group allocation to which one could be blinded.

Reporting for specific materials, systems and methods

We require information from authors about some types of materials, experimental systems and methods used in many studies. Here, indicate whether each material, system or method listed is relevant to your study. If you are not sure if a list item applies to your research, read the appropriate section before selecting a response.

Materials & experimental systems

n/a	Involved in the study	
x		Antibodies
x		Eukaryotic cell lines
x		Palaeontology and archaeology
	x	Animals and other organisms
x		Human research participants
x		Clinical data
x		Dual use research of concern

Methods

n/a	Involved in the study	
x		ChIP-seq
x		Flow cytometry
x		MRI-based neuroimaging

Animals and other organisms

Policy information about studies involving animals; ARRIVE guidelines recommended for reporting animal research

Laboratory animals	two male rhesus monkeys (10.5-11.0kg; 6-7 years old) were used in the study.
Wild animals	none.
Field-collected samples	none.
Ethics oversight	All procedures used in this study were approved by the Institutional Animal Care and Use Committee at Yale University, and conformed to the Public Health Service Policy on Human Care and Use of Laboratory Animals and Guide for the Care and Use of Laboratory Animals.

Note that full information on the approval of the study protocol must also be provided in the manuscript.
